# Negative Pressure Pulmonary Edema: A Case Report

**DOI:** 10.31729/jnma.4970

**Published:** 2020-07-31

**Authors:** Anisha Budhathoki, Yawen Wu

**Affiliations:** 1 Department of Anesthesiology, Third affiliated Hospital of Guangzhou Medical University, Guangzhou, Guangdong, China

**Keywords:** *extubation*, *laryngospasm*, *pulmonary edema*

## Abstract

Negative pressure pulmonary edema is an uncommon complication of the extubation of the endotracheal tube. An increase in intrathoracic pressure and negative pressure of the lung caused by acute laryngeal spasm results from acute upper respiratory obstruction causing life-threatening pulmonary edema by alveolar-capillary damage is called negative pressure pulmonary edema. We here describe 28-years old female case the preoperative diagnosis of pelvic inflammatory disease undergoing exploratory laporoscopy caused negative pressure pulmonary edema while extubation. With the immediate treatment, the patient was discharged without any abnormalities.

## INTRODUCTION

Negative pressure pulmonary edema (NPPE) is noncardiogenic pulmonary edema, induce due to an increase in intrathoracic pressure and negative pressure of the lung as a result of acute laryngeal spasm during extubation. NPPE is a life-threatening condition during the extubation after general anesthesia. The prevalence of NPPE occurs in 0.05% to 0.1%.^1^ It usually occurs in young healthy patients and during extubation due to which it is also called “post-extubation pulmonary edema.”^2^ We here discuss a case of 28 years old female case with a preoperative diagnosis of pelvic inflammatory disease occurring NPPE during extubation.

## CASE REPORT

A 28-years old lady presented to the third affiliated hospital of Guangzhou Medical University with complaints of severe abdominal pain in the lower abdomen and pelvic, fever, and pain during intercourse for 3 months. Normal past medical history without any comorbidity. There was no history of any past surgery, trauma, allergies, smoking, drug addiction, and infectious diseases. No specific family history was found.

On physical examination, there was tenderness in lower abdominal parts and pale skin. All the other preoperative assessments including laboratory examinations, imaging examinations, and physical examinations were under normal range.

She was scheduled for exploratory laparoscopy surgery with general anesthesia. The patient was fasted for 12 hours. American society of anesthesiologists (ASA) of degree I with height 155 cm, weight 70 kg was reported. Her Mallampati grade was II degree. Preoperative vital signs were stable with blood pressure of 110/60 mmHg, heart rate 60/m, and SpO_2_ 100%.

For Anesthesia induction drugs used sufentany l20 μg, propofol 120 mg, rocurium 50 mg, perecoxib 40 mg, remifentanyl 0.1 μg/kg/min continuous infusion, and desflurance 5% continuous infusion. Operation start time 8:45 am. Her vital signs during the operation were stable. EtCO_2_ was under normal range. The minimum alveolar concentration (MAC) was 6 to 6.2. Intraabdominal carbondioxide pressure was less than 15 mmHg. Rocurium 10 mg was given with 40 mins interval. Total operation duration was 2 hours. A total of 1500 mL of ringer lactate solution was administered during the surgery, the urine output was approximately 200 mL, and blood loss was less than 20 mL.

Extubation was done at 11:00 am with self spontaneous breathing observation, tidal volume of 400 L, and when she obeyed the command of opening eyes. Right after extubation patient had breathing difficulties, with mask ventilation positive pressure SpO_2_ was 88%. But it was ineffective, the further deterioration of SpO_2_ was observed with 75% to 60%. Pink frothy sputum was found around the mouth and during suction. Atropine 0.5 mg, neostigmine 1 mg was given. Bilateral pulmonary rales were auscultated at the time. With failure to raise in SpO_2_ patient was reintubated at 11:17 am with sevoflurance 1.8% continuous inhalation, fursemide 20 mg, methyprednisolone 80 mg, aminophyline 0.125 mg, dexmedetomide 20 ug, and ephidrine 6 mg was given. The SpO_2_ gradually returned to normal and the patient was taken to the Post-operative care unit (PACU) with an endotracheal tube. In PACU physical warmer, cis-atracurium 5 mg was used with continuous observation. The blood gas analysis and chest X-ray was taken immediately. The vital signs and urine output was normal in PACU. After 4 hours of observation, the patient was extubated successfully. The patient was sent to the ward without any discomfort and abnormalities.

**Figure 1.**
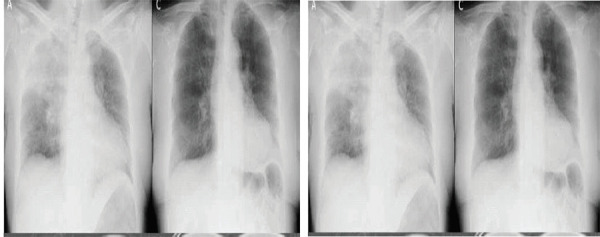
X-ray taken after reintubation and after 4 hours.

A chest X-ray is taken when the patient was sent to PACU and after 4 hours (Figure 1). The chest X-ray at the left side taken when the patient was sent to PACU showed bilateral diffuse, bilateral, hazy, interstitial opacity, pulmonary infiltration throughout both lungs. The chest X-ray at the right side taken after 4 hours patient was sent to PACU showed resolution of the process.

ABG at left side taken when the patient was sent to PACU and right side was taken after 4 hours showed improved SpO_2_ (Table 1).

**Table 1:** ABC taken after reintubation and after 4 hours.

Parameters	ABG at PACU	ABG after 4 hours
Base excess (BE)	1.0 mmol/L	1.0 mmol/L
Calcium (Ca)	1.10 mmol/L	1.10 mmol/L
Chlorine (Cl)	101 mEq/L	102 mEq/L
Glucose (Glu)	7.3 mmol/L	7 mmol/L
Haemoglobin (Hb)	123 g/dl	120 g/dl
Bicarbonate (Hco_3_)	24 mEq/L	23.3 mEq/L
Haematocrit (Hct)	36%	37%
Potassium (K)	3.86 mEq/L	3.96 mEq/L
Sodium (Na^+^)	140 mEq/L	143 mEq/L
Partial pressure of carbondioxide (PaCO_2_)	44 mmHg	42 mmHg
Acidity (pH)	7.32	7.35
Partial pressure of oxygen (PaO_2_)	60 mmHg	100 mmHg
Oxygen saturation (SPO_2_)	70%	97.6%

After the patient was sent to ward ABG report was found under normal ranges, timely vitals, and urine output observation was done. The blood gas analysis (ABG) and chest X-ray was taken regularly with no suspicion and abnormalities. Then the patient was discharged without any abnormalities after 3 days.

## DISCUSSION

Negative pressure pulmonary edema is an uncommon complication of the extubation of the endotracheal tube. NPPE has been reported mainly by anesthetists as a consequence of postoperative laryngospasm.^3^ The pathophysiology of NPPE begins with acute laryngeal spasm leading to severe upper airway obstruction. Intrathoracic pressure during acute upper airway obstruction is (> -100 cm H_2_O) whereas normal inspiratory chest pressure is (-2 to -5 cm H_2_O). The absolute value of intrathoracic transpulmonary negative pressure leads to the reduce of alveolar-capillary membrane pressure causing disruption of capillary barrier and alveolar epithelial barrier. All these causes exudation of blood vessels, which makes the red blood cells leak into the alveoli and causes significant lung or bronchial bleeding. These changes lead to red blood cell filtration into the lung and pulmonary edema.^4^ The increase in pulmonary blood volume occurs due to the increase in systemic arterial pressure secondary to the release of norepinephrine in response to hypoxia, hypercapnia, and agitation. Frothy pink pulmonary secretions is one of the most important syndrome to diagnose NPPE during extubation. And before making the diagnosis of NPPE, other causes of pulmonary edema particularly those requiring a rapid intervention (fluid maldistribution, anaphylaxis, and cardiogenic pulmonary edema), must be considered.^5^ The main diagnosis is done by incidence and symptoms, chest radiograph for pulmonary edema, and measure of pulmonary edema fluid/plasma protein ration. With proper treatment, NPPE can rapidly dissipate. The most important treatments include re-establishment of the airway by relief airway obstruction. Adequate oxygenation should be given, encourage the patient to cough after extubation. Application of positive airway pressure via face mask or LMA or Endotracheal intubation with ventilator support. Although NPPE does not result from fluid overload, it is recommended gentle diuresis using low-dose furosemide.^6^ The clinical syndromes can usually improves after 12-24 hours.^7^ But in our case, the symptoms were improved within 4 hours without any abnormalities. Patients with NPPE history should be kept in mind and prevention methods can be performed accordingly. It is also believed that the incidence of EPPN is higher than that found in the literature due to the fact that the cases are poorly diagnosed and, in many cases, confused with pulmonary edema due to fluid overload, aspiration pneumonitis and bronchospasm.^8^
